# Pseudophakic cystoid macular edema prevention and risk factors; prospective study with adjunctive once daily topical nepafenac 0.3% versus placebo

**DOI:** 10.1186/s12886-017-0405-7

**Published:** 2017-02-20

**Authors:** Sean McCafferty, April Harris, Corin Kew, Tala Kassm, Lisa Lane, Jason Levine, Meisha Raven

**Affiliations:** 1 Arizona Eye Consultants, 355 N. Silverbell Ave, Tucson, AZ 85745 USA; 2 Retina Associates of Tucson, 6561 E. Carondelet Dr, Tucson, AZ 85710 USA; 3 University of Arizona, 6422 E. Speedway Ave, Tucson, AZ 85710 USA

## Abstract

**Background:**

Define the effectiveness of a topical non-steroidal anti-inflammatory drug (NSAID) added to topical steroid use after uncomplicated phacoemulsification for the prevention of pseudophakic cystoid macular edema (PCME) using a prospective, randomized, double-masked, placebo-controlled clinical study.

**Methods:**

Eyes (1000) were randomized to placebo (497) or nepafenac 0.3% (503) used once daily, post-operatively for 5 weeks at two ophthalmology clinics. Diagnosis of PCME was made by clinical, ocular coherence tomography (OCT), and with fluorescein angiography confirmation. Correlation of PCME to NSAID use and the presence of pre-operative risk factors for PCME were assessed including, contralateral PCME, diabetic retinopathy, retinal vein occlusion, macular hole, epiretinal membrane, macular degeneration, retinal detachment repair, and prostaglandin use.

**Results:**

PCME was the most common complication associated with routine cataract surgery (4.2% with PCME risk factors, 2.0% with risk factors excluded). Topical nepafenac 0.3% significantly reduces the incidence of PCME compared to placebo when used after routine cataract surgery (*p* = .0001). When patients with pre-operative risk factors are excluded, the incidence of PCME between treatment and placebo groups is equivalent (*p* = 0.31). PCME relative risk (RR) was most significant in contralateral PCME (RR 19.5), diabetic retinopathy (RR 13.1), retinal vein occlusion (RR 12.9), macular hole (RR 7.7), and epiretinal membrane (RR 5.7). Prostaglandin use and previous retinal detachment were not shown to increase risk.

**Conclusion:**

Pseudophakic cystoid macular edema is common after phacoemulsification cataract surgery. Topical nepafenac 0.3% reduces PCME in patients with pre-operative risk factors for PCME compared to placebo but shows no benefit in patients without pre-operative risk factors.

**Trial registration:**

NIH ClincalTrials.gov retrospectively registered January 15, 2017, NCT03025945.

## Background

Cataract surgery is one of the most commonly performed surgeries. Although this procedure is generally safe, the most common adverse event leading to postoperative vision loss is the development of pseudophakic cystoid macular edema (PCME) [1]. The incidence of clinically significant acute PCME (vision loss greater than 20/40 from an expected 20/20, or equivalent) ranges from 0.6 to 3.6% with a peak incidence at around 5 weeks after uncomplicated cataract surgery [1–3]. The majority of PCME cases resolve spontaneously with vision returning to normal [1, 2].

Clinically significant PCME includes ophthalmic and angiographic findings with vision loss. A visual acuity of 20/40 best corrected visual acuity (BCVA) or less has been considered clinically significant by most studies [4, 5]. The gold standard for identifying PCME has been with fluorescein angiography and has yielded prevalences as high as 9.1 to 25.5% [2]. Findings of angiographic PCME do not correlate well with vision loss. However, measurement of macular thickness with optical coherence tomography (OCT) does correlate well with visual impairment [6, 7].

The prevalent use of NSAIDs following cataract surgery has evolved from the surgeons desire to reduce the incidence of PCME which is the leading cause of decreased visual acuity following an uneventful phaco-emulsification cataract surgery [3]. Our unpublished survey of 62 cataract surgeons indicates that 72% of those surveyed use topical NSAIDs primarily to prevent PCME [Arizona Surgical Eye Study: Survey of 62 U.S. cataract surgeons regarding their use of topical NSAID’s following cataract surgery May to July 2014, unpublished]. No ophthalmic NSAID has a clinical indication for this use. Many studies have shown a decrease in PCME from an angiographic or OCT basis but not using a prospective design examining clinically significant loss of vision [2, 3, 6, 8–10]. Also, no prospective studies demonstrate significant differences in the incidence of PCME with the use of NSAIDs, citing insufficient sample size [4, 5, 11, 12].

There is evidence that topical NSAIDs reduce early post-operative anterior segment inflammation [13, 14]. It remains unknown whether NSAID use for four (4) weeks translates into improved outcomes or overall comfort of the patient [13, 14]. Studies have linked other benefits such as decreased capsular phimosis and miosis [15].

PCME is thought to be due to disruption of the blood aqueous and blood retinal barrier [16]. Although PCME can occur in healthy eyes with no surgical complications, risk factors increase the likelihood of it occurring. Risk factors include PCME in the contralateral eye and anything that may disrupt the blood retinal barrier such as diabetes mellitus, uveitis, retinal vein occlusion, retinal degeneration, macular degeneration, radiation retinopathy, epiretinal membranes, choroidal tumors, prostaglandin analog use, and aging [1]. Because PCME is largely thought to be caused by an increase in prostaglandins, NSAIDs are routinely used in the postoperative period to inhibit cyclooxygenase (COX-1 and COX-2) enzymes, which prevent the production of prostaglandins and their downstream inflammatory effects [17–19].

Almeida et al. and Tzelikis et al. have prospectively demonstrated negligible benefit with NSAID use after uncomplicated cataract surgery in patients without risk factors [4, 20]. Some literature supports the use of NSAIDs in the treatment and prophylaxis of PCME in uncomplicated surgical cases. Patients were compared taking diclofenac 0.1% with patients taking fluorometholone 0.1% as well as those taking prostaglandin analogs and found that overall incidence of angiographic PCME measured 5 weeks postoperatively was 54.7% in the steroid group and 5.7% in the NSAID group [21]. Currently, there is insufficient data defining the NSAID role in treating acute PCME resulting in no standard protocol at this time [22].

The presented independent, randomized, double blind, prospective study evaluates the efficacy of post-operative topical NSAIDs over a large sample size to prevent acute PCME for routine cataract surgery. Included are the common risk factors seen in clinical practice such as contralateral PCME, prostaglandin analog use, diabetic retinopathy, epiretinal membrane, macular degeneration, previous retinal surgery, and retinal vein occlusion.

## Methods

A prospective, randomized, double-masked, placebo-controlled clinical trial was performed at two sites in Tucson Arizona, as part of the Arizona Surgical Eye Study (ASES). The ASES was an independent prospective randomized study designed to examine the incidence and causation of post cataract surgical complications conducted from 10–15-2013 to 11–1–2015 stemming from NSAID use, phacoemulsification machine use, IOL type, and identified risk factors. The ASES specifically examined complication type and incidence, ocular discomfort, inflammation, capsular phimosis, endothelial cell count change, and posterior capsule opacification postoperatively. The clinical evaluation described herein is a subset of the available data at the time of publication examining solely the incidence of PCME. The clinical trial was approved by Western Independent Review Board. All patients were treated according to the Declaration of Helsinki document on human research ethics, and underwent informed consent.

### Study protocol

Subjects, 18 and older, were enrolled from the clinic. The planned enrollment was 970 eyes (1000 completed) for probable statistical significance with a 1:1 ratio of control: treatment. Subjects were chosen from patients who had visually significant cataracts and were to undergo phacoemulsification with implantation of an intracapsular positioned intraocular lens. Subject’s eyes individually were randomly assigned by the compounding pharmacy using random number generator in groups of 10 to receive a placebo of sterile saline drops or nepafenac 0.3%. Both study drops (nepafenac and placebo) were produced individually by the pharmacy in a generic bottle marked by a code and instructions for use. The codes were maintained and utilized solely by the pharmacy to determine the content of the bottle revealed following the completion of the study. All patients received topical prednisolone 1% four times daily for the first week, tapered to 2 times daily over the second week and 1 time daily for the subsequent 3 weeks which approximates most common practice [1, 3, 4]. No additional steroids were used intraoperatively. Ofloxacin 0.3% was used 4 times daily for the first week and discontinued.

In contrast to other studies, patients included those on prostaglandins or on glaucoma medications, those with an epiretinal membrane, macular degeneration, or diabetes mellitus (with or without retinopathy), macular hole, previous retinal surgery, and history of central or branch retinal vein occlusion. Glaucoma medication patients were included as a risk factor if the medications were used any time prior to the surgery. Epiretinal membranes were identified and included if seen on funduscopic exam or OCT prior to cataract surgery within the macula (central 5.5 mm diameter around the fovea or 2.75 disk diameters). Diabetic retinopathy was included if there was any demonstrated evidence upon funduscopic exam. Previous retinal surgery included any patient receiving any vitreoretinal procedure excluding laser procedures. Macular holes, central and branch retinal vein occlusions were included regardless of stage. Exclusion criteria included previous uveitis (<1 year), previous anterior segment intraocular surgery or a hypersensitivity or allergy to NSAIDs. Any patients who had a complicated cataract surgery (posterior capsule rupture, vitreous loss, retained cortical material, significant corneal edema or an IOL not placed in the capsular bag) were excluded from the data analysis.

During the patient’s initial visit, information on demographics was obtained, including age, sex, birth date, ethnicity, as well as ocular history and medical history. The baseline exam included intraocular pressure, dilated fundus exam, slitlamp exam and best-corrected visual acuity (BCVA) by Snellen chart. Also, before surgery, a trained technician performed a baseline OCT macular cube scan (OCT protocol described below).

Three surgeons participated, LL, SM, and JL, with similar methods and amount of time performing the surgery. The surgeries were phacoemulsification cataract extraction with posterior chamber in-the-bag IOL placement. An equal number of patients (500 in each group) were randomized in groups of 10 and underwent phacoemulsification cataract surgery with an Alcon (Fort Worth, TX) Infinity machine using an SA60AT IOL and an Abbott Medical Optics (Santa Ana, CA) WhiteStar Signature using a ZCB00 IOL. Both the NSAID treatment group and control group were equally divided among the two phacoemulsification machines and their respective IOL’s which was confirmed following the study. Postoperatively, patients were routinely followed up on day one, at 1 week and at 5 weeks. All visits included a BCVA, slit lamp exam, IOP, and an OCT macular cube scan. Subjects were assessed at all visits for any postoperative inflammation, capsular phimosis and posterior capsule opacification. During the study, patients were interviewed about any issues or concerns they had during that postoperative period including medicine cost [19]. Any adverse events or complications were followed closely and treated during the post-operative period.

Once the two groups were randomized, one group received one drop a day of nepafenac 0.3% for 5 weeks which is the recommended dosage for the new higher concentration nepafenac. Nepafenac ophthalmic suspension 0.3% was supplied as a sterile, aqueous suspension with a pH approximately of 6.8. The osmolality of the nepafenac 0.3% is approximately 300 mOsm/kg. Nepafenac 0.3% contains: Active: nepafenac 0.3% Inactive: boric acid, propylene glycol, carbomer 974P, sodium chloride, guar gum, carboxymethylcellulose sodium, edentate disodium, benzalkonium chloride 0.005%. The topical NSAID was begun on the first post-operative day of surgery. The other group received the placebo which were buffered sterile saline drops with a pH approximately of 7.0 and osmolality of 290 mOsm/kg, dosed at the same frequency. The placebo drops ingredients included: Sodium chloride; Boric acid; calcium chloride; magnesium chloride; potassium chloride; purified water; sodium borate; and carboxymethylcellulose sodium. The pharmacy provided identical opaque generic bottles that were coded with identification numbers only; no drug information was visible on the bottle. The bottles also had a patient identification number and expiration date displayed. Only the pharmacy retained the information for the codes and this information was not shared with investigators until completion of the study. All participants of the study also received prednisolone acetate 1% suspension 4 times a day for 1 week, followed by 2 times a day for 1 week, followed by 1 time daily for 3 weeks. These medications were started on the day after the surgery. Ofloxacin 0.3% drops were administered to all participants and dosed at four times a day, starting one day before surgery until 1 week after surgery.

### Optical coherence tomography

OCT was performed on all subjects pre-operatively, at 1 week and at 5 weeks postoperatively. A spectral-domain Cirrus HD-OCT device was used by experienced staff who were also masked to treatment. The imaging protocol utilized the OCT to measure macular volume (VOL, mm [3]), central subfield thickness (CST, μm) and average macular thickness (AVG,μm). For each subject, the best-quality macular images of all scans were chosen and unreliable scans were excluded.

### Study endpoint

Post-operative clinical findings of PCME within 6 weeks including symptomatic or decreased BCVA and angiographic as well as OCT corroboration to confirm the presence of PCME with experienced vitreo-retinal surgeons. Clinically significant PCME was defined as including both: 1. A loss of two (2) lines of best corrected visual acuity (BCVA) from the expected post cataract BCVA (example: 20/40 BCVA from an expected 20/20 BCVA) or visually symptomatic distortion. 2. OCT, clinical, and angiographic demonstrated PCME. All cases of PCME were successfully treated by the retinal surgeon with continuation of drops and subtenons injections of steroids. Secondary outcome measurements included ocular discomfort, inflammation as well as the comparison of ophthalmic medications for tolerability (COMTOL) questionnaire analysis. The intent-to-treat group consisted of subjects who completed surgery, received the study drugs and completed the follow-up. However, any and all patients who received the study drugs were included in the safety analysis. Complications and adverse events were documented when interviewing the participants or recorded by investigators. Adverse events, for the purpose of this study, were defined as any unintended or unwanted sign, symptom or clinical result that could be associated with the study drugs.

### Statistical analysis

The sample size using the Wald method was determined prior to the study at approximately 485 in each group. Given the likely incidence of clinically significant PCME between 1 and 3% this sample size would likely lead to a statistically significant outcome [18]. If no statistical difference was seen at this sample size, then it would be expected that a routine cataract surgeon would not see any difference in his patient outcomes in over 2 years of surgery (@500 cases/year) [Arizona Surgical Eye Study: Survey of 62 U.S. cataract surgeons regarding their use of topical NSAID’s following cataract surgery May to July 2014, unpublished]. A 35% increase in macular volume is considered to be significant by a previous study and was used as our threshold for the OCT measures [10]. Comparison of OCT’s between five (5) weeks and baseline macular volumes were performed within subjects using a 2-way ANOVA (*p* = .05) between subjects in placebo and treatments groups. Two additional 2-way ANOVAs were also completed within subjects pre-op and 5-week OCTs between placebo and treatment groups, both with and without risk factors. As mentioned previously, a loss of more than 2 lines of expected BCVA with angiographic evidence of CME was used to define “clinically significant” PCME. The incidence of clinically significant PCME with both NSAID nepafenac 0.3% treatment and control was determined and compared with Chi-squared (Dof = 1) tests with all patients and both including and excluding risk factors. An odds ratio or relative risk of probable PCME was calculated for each of the identified risk factors by comparing the ratio of the incidence of PCME found with each risk factor to the baseline incidence of PCME without risk factors. Lastly, a logistical regression analysis was completed to examine the probability of multiple other factors (such as IOL type, phaco machine used) affecting the outcome of the study.

## Results

Six hundred and sixty two (662) patients, one thousand eyes (1000), completed the study (1007 eyes enrolled). Two patients had intraoperative complications and were withdrawn from the study. One patient withdrew voluntarily due to drop intolerance. Four patients were lost to follow-up.

Pseudophakic cystoid macular edema (PCME) was the most common complication following cataract surgery at 4.2% with careful diagnosis. The Incidence of PCME was 2.1% with pre-operative risk factors excluded. All cases of PCME resolved with treatment. The next highest pseudoemulsification related complications were persistent iritis (0.4%), posterior capsular rupture (0.2% - excluded from this study) and persistent corneal edema (0.1%). Complications did not statistically correlate with PCME in this study. Five hundred three (503) patients had cataract surgery with an Alcon Infinity pseudoemulsification machine and a SA60AT implant (Ft. Worth, TX). Four hundred ninety seven (497) patients had cataract surgery with an Abbott Medical Optics WhiteStar Signature pseudoemulsification machine and a ZCB00 implant (Irvine, CA). The incidence if PCME was 21 in each surgical equipment/implant group. Risk factors were also equally divided in the groups.

Results of PCME between nepafenac 0.3% and placebo groups using the Chi-squared tests were subdivided into all patients, those with risk factors and those without risk factors. Table 1 shows the results of the PCME Chi-squared probable significance test.

**Table 1:** PCME incidence Chi-squared significance

Patient group	Patients	PCME	Chi- Squared (*p*=)
(*n* = 1000)	Number of eyes	Percent incidence	NSAID use null probable significance (*p*<0.05)
All patients	1000	4.20	0.0001
Patients with Risk factors	308	8.77	0.00003
Patients without Risk factors	692	2.17	0.31089

**Fig. 1:**
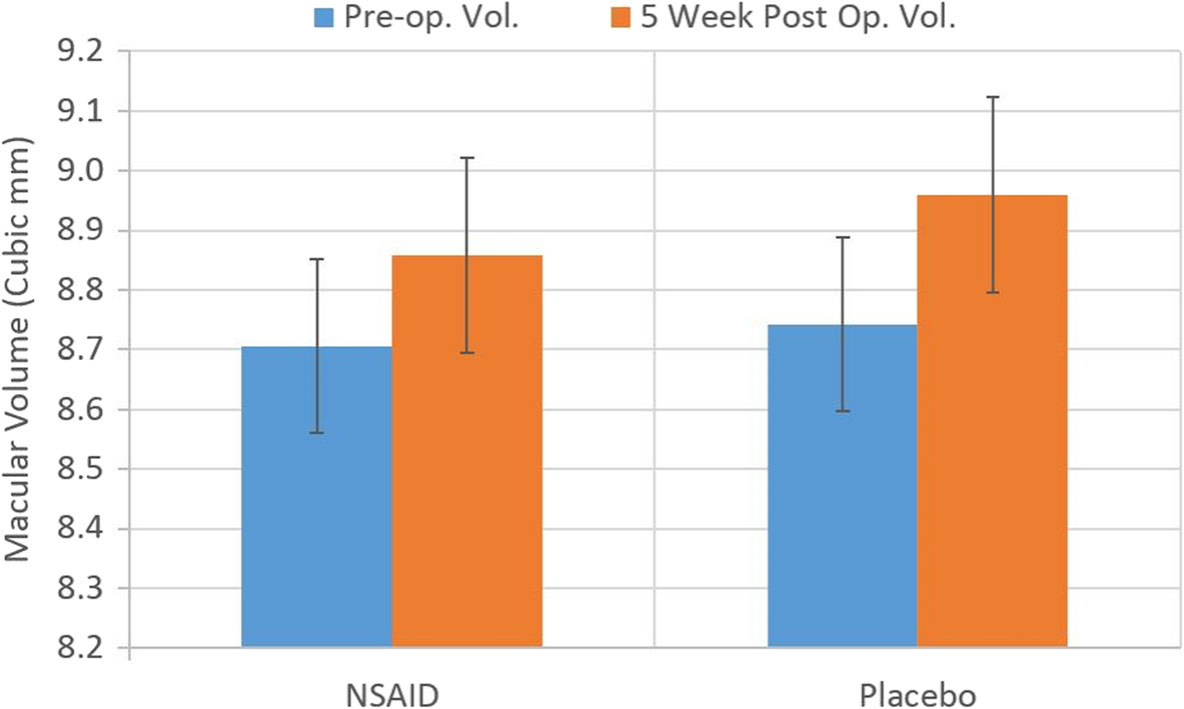
Post-operative change in macular volume in patients without risk factors (95% CI)

**Fig. 2:**
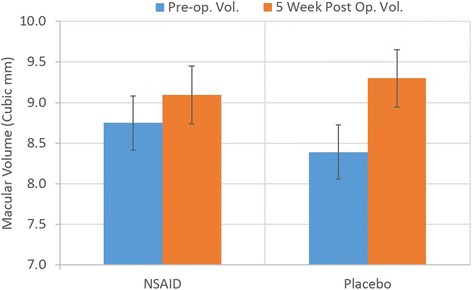
Post-operative change in macular volume in patients with risk factors (95% CI)

Comparison of macular volume was completed with OCT measurement pre-op to 5 weeks post-op examining nepafenac 0.3% and with placebo groups. The 2-Way ANOVA in Table 2 indicates significantly increased macular volume post-operatively in all patients. Treatment with nepafenac 0.3% when compared to placebo was only statistically significant in patients who had risk factors. Figures 1 and 2 depict the changes in post-operative macular volume from the pre-operative measurement comparing the use of nepafenac 0.3% to placebo with a 95% confidence interval. This also confirms the findings from the 2-way ANOVA tests.

**Table 2:** 2 Way ANOVA examining intra-subject macular volume change pre-op and 5 weeks post-op, also between NSAID and placebo groups with all patients and with those having risk factors

	2-Way ANOVA with replication
All patients macular volume	*P-value*
Post-operative volume change	0.032
NSAID vs. placebo volume change	0.858
Patients with Risk Factors macular vol.	*P-value*
Post-operative volume change	0.003
NSAID vs. placebo volume change	0.031

The relative risk of developing PCME was calculated for each of the eight identified risk factors to quantify their significance in terms of probable PCME for the clinician. Each risk factor was statistically equally distributed between the nepafenac and placebo groups. Table 3 illustrates the incidence of PCME with each risk factor and relative risk in terms of an odds ratio. The highest risk groups were those with PCME in the previously operated eye, those with diabetic retinopathy, and those with a vein occlusion. Interestingly both macular degeneration and prostaglandin use showed a negligible increased risk of developing PCME. No patients with previous retinal detachment repair developed PCME.

**Table 3:** Risk factor odds ratio (relative risk) of developing PCME

Risk factor odds ratio	PCME	PCME	Odds ratio
Patient group (*n* = 47)	Number of eyes	Percent incidence	Relative risk compared to no risk factor (95% CI)
PCME Contralateral Eye	11	42.3	19.5 (18.5–20.6)
Diabetic Retinopathy	19	28.4	13.1 (12.3–13.9)
Vein Occlusion CRVO/BRVO	3	27.3	12.6 (9.0–16.2)
Macular Hole	3	16.7	7.7 (5.7–9.7)
Epiretinal Membrane	7	12.3	5.6 (4.7–6.8)
Prostaglandin Use	1	3.6	1.6 (0.1–3.16)
Macular Degeneration	3	3.1	1.4 (0.6–1.8)
All Risk factors	42	13.6	6.3 (4.9–7.6)

Additional PCME contributing factors were analyzed using a binary logistical regression. Factors included: Gender, age, ethnicity, IOL type, phacoemulsification machine, NSAID use, and Risk factor presence. The results confirmed the previous findings in that the predominant factors were pre-operative risk factors followed by nepafenac use. NSAID use and presence of a pre-operative risk factor significantly outweighed the other potential factors. Table 4 are the calculated binary logistical regression coefficients for each factor.

**Table 4:** Binary logistical regression factor coefficients for possible PCME contributing factors

Factor	Coefficient	Value
IOL/Phaco Machine	b1	0.19
NSAID use	b2	1.32
Risk Factor presence	b3	1.69
Gender	b4	0.11
Ethnicity	b5	0.32
Age	b6	0.29

## Discussion

The new higher concentration topical nepafenac 0.3% dosed once daily reduces PCME in patients with pre-operative risk factors for PCME compared with placebo but shows no benefit in patients without pre-operative risk factors. Pseudophakic cystoid macular edema is common after phacoemulsification cataract surgery and its prevention is the practitioner’s primary reason for post-operative NSAID use [Arizona Surgical Eye Study: Survey of 62 U.S. cataract surgeons regarding their use of topical NSAID’s following cataract surgery May to July 2014, unpublished]. There is a potential for reduced NSAID utilization if its use is limited to cataract surgery patients with pre-operative risk factors for PCME as identified by their relative risk.

While the present independent study examined nepafenac 0.3% dosed once daily, it is reasonable that the results can be extrapolated to topical NSAID’s in general. The findings are consistent with other studies examining other topical NSAIDs reducing the incidence of macular thickening compared to placebo [4–6].

This study is part of the larger Arizona Surgical Eye Study (ASES) which also examines the other post-operative uses of nepafenac 0.3% as well as surgical equipment, IOL type and complications. These other results were not included in this publication except to insure they did not have an effect on PCME. The negligible effect on PCME by other variables in the ASES study was demonstrated by the binary logistical regression analysis. There may be other combined effects of nepafenac which make its routine use beneficial beyond the development of PCME, such as its clinical indication of reducing post-operative inflammation.

A higher than expected incidence of PCME was noted at 4.2%. The authors see this as a result of the high incidence of risk factors, approximately 30%, and the inclusion of pre-operative risk factors in the study [18]. If risk factors are excluded the incidence of PCME is 2.1% which is consistent with previous studies. The relative risk of PCME with pre-operative risk factors is also consistent with a recent large retrospective study [23]. The presence of increased macular volume post-operatively also is consistent with previous studies [4].

The new higher concentration nepafenac 0.3% dosed once daily, appears well tolerated. No adverse events were found with the medication even at its higher concentration (0.3%) and it was found to be safe for routine use in post-operative pseudoemulsification patients. These findings are similar to other studies examining the lower dose nepafenac 0.1% administered 3 and 4 times daily [17, 18].

The continued ASES study may substantiate the use of NSAIDs adjunctively in reducing inflammation in the first post-operative week [13, 14]. Anti-inflammatory benefit may exist beyond the first week but this remains to be determined by subsequent analysis of the collected data.

## Conclusions

The results of this non-industry supported, large, prospective, double-blind study assist the practicing ophthalmologist in deciding the appropriate use of topical NSAIDs in the prevention of PCME. Based upon the study’s results, the ophthalmic community, with discretion, could elect to alter the present practice patterns maintaining or improving patient outcomes while potentially reducing NSAID use.

## Abbreviations

ASES: Arizona Surgical Eye Study
BCVA: Best corrected visual acuity
IOL: Intraocular lens
NSAID: Non-steroidal anti-inflammatory drug
OCT: Optical coherence tomography
PCME: Pseudophakic cystoid macular edema
RR: Relative risk
